# Periodontal disease and chronic kidney disease: mechanistic insights and novel therapeutic perspectives

**DOI:** 10.3389/fcimb.2025.1611097

**Published:** 2025-06-25

**Authors:** Shuxin Li, Hongliang Cao, Yueqiu Zhang, Fulin Wang, Gengchen Huang, Binbin Wang, Wei Wei, Gang Wang

**Affiliations:** ^1^ Department of Urology, The First Hospital of Jilin University, Changchun, China; ^2^ Department of Otorhinolaryngology, Head and Neck Surgery, West China Hospital, Sichuan University, Chengdu, China

**Keywords:** periodontal disease, oral microbiota, chronic kidney disease, systemic inflammation, inflammatory factors, reactive oxygen species, therapy

## Abstract

Periodontal disease (PD) is one of the most common chronic diseases of the oral cavity, and it usually refers to chronic inflammatory diseases caused by the infection of pathogenic microorganisms in the oral cavity. PD mainly affects the tissues around the teeth, causing inflammation of the gums and periodontium, destroying the alveolar bone, and even leading to tooth loosening. Many studies have shown that PD is not only limited to oral health but is also associated with diseases of multiple systems throughout the body. In recent years, more and more studies have focused on the interaction between PD and urologic diseases, especially chronic kidney disease (CKD). PD is also a typical oral disease frequently observed in patients with kidney disease. This paper reviews the potential link between PD and CKD and discusses the interaction’s pathologic mechanisms and clinical implications. In addition, this review aims to identify possible pathogenic mechanisms and suggest potential ways to target PD to prevent and treat CKD. This would lead to better treatment options to delay the progression of CKD.

## 1 Introduction

Chronic kidney disease (CKD) is a common disease of the urinary system, with an average population prevalence of CKD of about 10 percent, with women more likely to suffer from the disease than men (Suriyong et al., 2022; Li et al., 2024). Most patients worldwide reside in low and middle-income countries, with China and India accounting for about one-third of all cases worldwide (Butt et al., 2022; Suriyong et al., 2022). In Europe and North America, the prevalence of kidney failure is low. However, the global incidence of CKD has continued to rise in recent years as the proportion of the worldwide population that is aging increases and the prevalence of diabetes and obesity in the population grows (van Rijn et al., 2020; Ghassabi et al., 2022; Hofherr et al., 2022; Worthington et al., 2024). This places a substantial economic burden on society and hurts patient’s quality of life and survival. CKD is an abnormality in the kidneys’ structure and function lasting over 3 months (Ortiz et al., 2022). According to the kidney disease: Improving Global Outcomes (KDIGO) classification, CKD is categorized into stages G1-G5. The clinical manifestations of patients in different stages of CKD are various. Clinical symptoms are not apparent in the early stage, occasionally accompanied by high blood pressure, mild edema, and increased nocturia (Zoccali et al., 2023; Wang et al., 2025). In the middle stage of CKD, multi-systemic symptoms gradually appear, and patients often feel generalized fatigue and weakness, loss of appetite and weight loss, gastrointestinal symptoms, and gastrointestinal bleeding tendency (Rhee et al., 2022; Zoccali et al., 2023). Symptoms related to the accumulation of metabolic waste and disturbance of the internal environment occur during disease progression, such as generalized edema caused by sodium retention, cardiac arrhythmia caused by hyperkalemia, and limb weakness caused by hypocalcemia (Kalantar-Zadeh et al., 2022). The kidneys are also responsible for the synthesis and secretion of various hormones. CKD disrupts the endocrine function of the kidneys, resulting in disorders of glucose and lipid metabolism and erectile dysfunction in men, and the impaired synthesis of erythropoietin (EPO) and activated vitamin D leads to the development of renal anemia and renal bone disease (Kalantar-Zadeh et al., 2022; Ku et al., 2023). When CKD reaches the end stage, severe uremic symptoms can appear, with pericarditis, acute pulmonary edema, severe anemia, and uremic encephalopathy (Kalantar-Zadeh et al., 2022; Frąk et al., 2024).

Periodontal disease (PD) is a common oral disease that refers to the inflammation of the supporting tissues around the teeth, mainly including gingivitis or periodontitis (Zhao et al., 2024). The periodontal supporting tissues primarily include the gums, periodontal ligament, cementum, and alveolar bone. PD is a complex inflammatory response caused by oral dysbiosis, associated with an immune reaction to microorganisms and their toxic products/wastes (Zhang et al., 2025). Many studies have shown that PD is not limited to the oral cavity but is potentially related to developing a wide range of systemic diseases (Mukherjee et al., 2025). Various microorganisms, such as bacteria, fungi, viruses, and protozoa, are present in the human oral cavity and are referred to as the oral microbiota. A normal microbiota maintains the stability of the oral environment, maintains normal oral pH, and stimulates the oral mucosa to activate immunoprotected functions (Chattopadhyay et al., 2023). However, these microorganisms can also cause oral diseases, and an increase in pathogenic bacteria and their metabolites can lead to tooth destruction and inflammation of tissues in the oral cavity (Zhang et al., 2025). Furthermore, oral microorganisms can affect multiple systems throughout the body by entering the bloodstream and producing inflammatory factors (Li et al., 2022a). In recent years, several studies have shown that PD caused by oral microbiota disorders plays a role in the development of kidney diseases. Therefore, when treating patients with PD, we should be alert to the deterioration of renal function. In this review, we summarize the epidemiological and pathogenic mechanism studies between PD and CKD to explore potential approaches to preventing and treating CKD through intervention in PD.

## 2 Observational and experimental evidence suggest a strong association between PD and CKD

Recent observational studies have shown that periodontal health and oral microflora in patients with CKD significantly differ from those in healthy men (Kaymaz et al., 2024). This suggests that PD may play a role in the development, progression, and altered treatment outcomes of CKD. In addition, several animal studies have reported similar findings (Kaymaz et al., 2024).

### 2.1 Bidirectional association between PD and CKD

There is a significant bidirectional association between PD and CKD, with periodontitis exacerbating CKD progression through systemic inflammation and oxidative stress. At the same time, immune dysregulation and metabolic abnormalities in patients with CKD may also exacerbate periodontitis. A structural equation modeling study by Pravin-Sharma et al. confirmed that each 10% increase in periodontal inflammation leads to a 3% decrease in renal function, and each 10% decrease in renal function exacerbates periodontal inflammation by 25% (Sharma et al., 2021). As a chronic inflammatory disease, periodontitis has pathogenic bacteria and inflammatory mediators that can enter the systemic circulation through ulcers in the oral mucosa, inducing a systemic inflammatory response affecting CKD. In a cross-sectional study of 998 elderly Japanese individuals, a significant positive association was found between early and advanced periodontitis and CKD, and this association was more pronounced in those with the presence of atherosclerosis (Shimizu et al., 2023).In another clinical controlled study, a higher prevalence of PD was found in patients with CKD by comparing their periodontal health with that of healthy controls (Ma et al., 2021). Gingival crevicular fluid (GCF) and serum levels of inflammatory markers CRP and interleukin-8 were also recorded, and patients with end-stage renal disease were found to have a higher prevalence of inflammatory and metabolic markers associated with periodontal health than healthy patients (Ma et al., 2021).In addition, in a retrospective cohort study of patients with type 2 diabetes mellitus, it was found that patients who received periodontal care had a 32-44% lower risk of dialysis initiation compared to those who did not receive periodontal care (Kusama et al., 2025). A cross-sectional study in Japan, in which questionnaires were used to record the number of teeth in 16,128 participants and the maintenance of dialysis treatment in patients with end-stage renal disease, found that tooth loss may be associated with declining renal function in Japanese men younger than 65 years of age (Kondo et al., 2024). Amr Saeed Ghanem also found that C-reactive protein and glomerular filtration rate can be potential markers for predicting periodontitis and that early intervention treatment can prevent systemic complications (Ghanem, 2025). This suggests that treatment of PD may impact the development of CKD. These observational studies found significant differences in periodontal health in CKD patients compared to healthy patients. Healthy periodontal condition was associated with a lower risk of developing CKD.

### 2.2 Specific associations between PD and renal transplant patients

Patients with end-stage CKD need kidney transplantation to cure the disease and improve their quality of life ultimately. PD is associated with the development of complications after renal transplantation. Cardiovascular disease is the leading cause of kidney transplant failure. PD may further exacerbate the risk of cardiovascular disease by causing a systemic inflammatory response, leading to kidney transplant failure (Wynimko et al., 2020; Aziz et al., 2022). A clinical controlled study by Marcela A. Santos-Paul et al. found that periodontal therapy improves adverse cardiovascular events in CKD by providing 24-month outcome follow-up of CKD patients treated with or without periodontal treatment (Santos-Paul et al., 2019). Infection is also one of the leading causes of postoperative mortality in kidney transplant patients; periodontal disease may lead to the formation of localized foci of infection in the oral cavity, where bacteria can spread throughout the body via the bloodstream and increase the risk of infection after kidney transplantation. PD may be considered a nontraditional risk factor in the preoperative evaluation for kidney transplantation (Chan et al., 2021). In a controlled study that included 77 patients, participants in the oral infection group were found to have a significantly higher risk of infection-related complications at 100 days after kidney transplantation than those in the oral health group, and the difference was statistically significant (Pol et al., 2022). By comparing the periodontal health status of 53 renal patients before and after kidney transplantation, Karita Nylund et al. found that the periodontal status of patients after kidney transplantation was better than before dialysis (Nylund et al., 2018). This finding confirms the importance of treating oral infectious lesions in the pre-dialysis phase to prevent adverse outcomes after kidney transplantation. PD may also be associated with decreased postoperative transplant renal function. One study found that kidney transplant recipients with higher levels of periodontal inflammation had a relatively higher risk of worsening kidney function (Sharma et al., 2021). This may be due to the inflammation and oxidative stress triggered by PD-causing damage to the renal microvasculature and glomeruli, affecting the filtration and metabolic functions of the kidney (Chapple et al., 2025). The association of PD with elevated blood glucose after kidney transplantation was found in a cross-sectional study, suggesting an association of PD with metabolic disturbances after kidney transplantation (Gomes et al., 2022). In some studies, PD has been recognized as a CKD complication, where CKD alters the biochemical composition of saliva and induces progressive ecological dysregulation of the oral microbiota, with a decrease in the abundance of healthy oral microorganisms and an increase in pathogenic bacteria (Randall et al., 2025). Dysbiosis of the oral microflora is thought to be the initiating link in CKD-induced PD (Randall et al., 2025). For patients with CKD, oral health education and screening and treatment of PD should be strengthened before kidney transplantation to reduce the risk of postoperative complications.

## 3 Potential mechanisms of interaction between multiple aspects of PD and CKD

**Figure 1:**
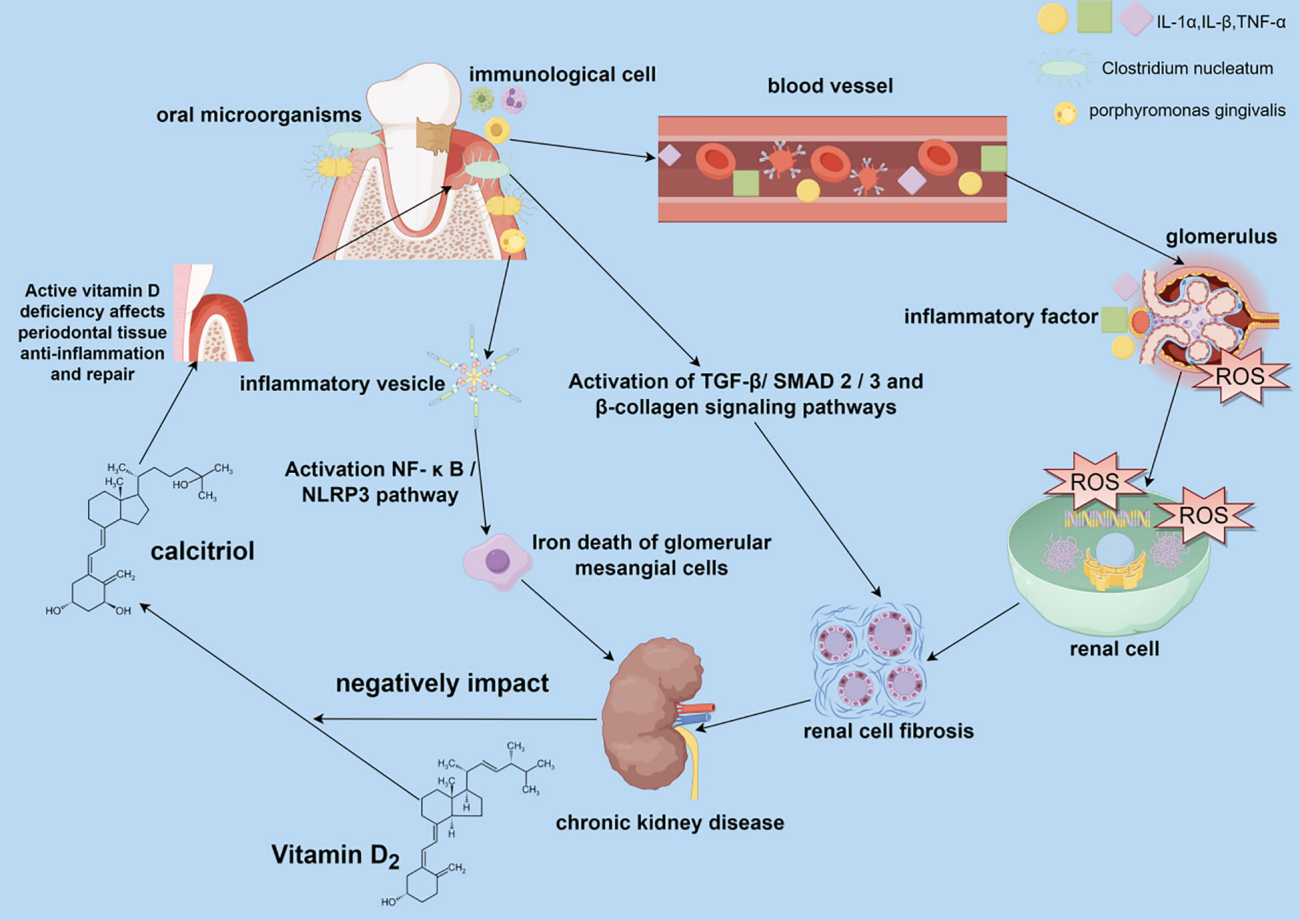
Impact of periodontal disease (PD) on the pathogenesis of chronic kidney disease (CKD). PD caused by oral dysbiosis can affect renal health in both direct and indirect ways. Oral pathogenic bacteria can be transferred to the kidneys via the bloodstream and contribute to renal pathology, causing renal cell fibrosis and iron death of glomerular mesangial cells. Inflammatory factors and other immune molecules produced by the immune cell infiltration of oral periodontitis are transferred to the kidneys through the bloodstream, causing changes in the local immune-inflammatory environment of the kidneys, making a large amount of reactive oxygen species that damage the renal cell membranes and DNA and proteins within the renal cells, and formation of renal fibrosis in the process of self-repair of the damaged renal cells, which leads to the development of CKD.

Although current observational studies have pointed to several associations between PD and CKD, and the differences in characterization in these studies have the potential to be targeted for prevention and treatment, the exact mechanisms of their effects still need to be fully clarified. This section describes in detail the potential mechanisms of action between PD and CKD and how PD may influence the development of CKD. There is a complex bidirectional association between PD and CKD, and the mechanisms of interaction involve inflammation, oxidative stress, oral microbial imbalance, metabolic disorders, and immune regulation (**Figure 1**).

### 3.1 PD causes inflammation and oxidative stress to damage the kidneys

PD is a chronic infection; its pathogenic bacteria, such as *porphyromonas gingivalis* and its metabolite lipopolysaccharide, can enter the blood circulation through the ulcerated gingival mucosa and activate the systemic immune system to cause systemic inflammation and oxidative stress to damage the kidneys (Chopra and Sivaraman, 2019). Moreover, periodontal plaque can cause local chronic inflammation, leading to immune cell infiltration and the release of many inflammatory factors, such as interleukin-1 (IL-1) and tumor necrosis factor-alpha (TNF-α) (Chen et al., 2024). These inflammatory mediators can diffuse throughout the body via blood circulation, triggering a systemic inflammatory response and oxidative stress on the kidneys (Chen et al., 2024). Oxidative stress upregulates NOD-like receptor family pyrin domain containing 3 (NLRP3) inflammatory vesicles and promotes the release of pro-inflammatory factors such as interleukin-1β (IL-1β), creating a vicious cycle of oxidative stress-inflammation-renal fibrosis (Colombo et al., 2020; Hickey et al., 2020). Inflammatory mediators in the systemic inflammatory response can act directly on renal tissues, activating glomerular mesangial cells and tubular epithelial cells, causing them to express inflammation-related genes and release more inflammatory factors, triggering a localized inflammatory response in the kidneys (Parsegian et al., 2022). In addition, these inflammatory mediators can induce an imbalance between the proliferation and apoptosis of renal cells, promoting glomerulosclerosis and tubulointerstitial fibrosis, leading to impaired renal structure and function (Cohen-Hagai et al., 2023). During periodontal inflammation affecting the kidneys, inflammatory factors such as macrophages can activate immune cells in the kidneys. During bacterial clearance, the body’s immune cells produce large amounts of reactive oxygen species (ROS), such as superoxide anion and hydrogen peroxide. Under normal conditions, the body’s antioxidant system scavenges the excess ROS. However, superoxide dismutase activity is reduced in patients with PD and CKD. Local production of ROS in PD and systemic oxidative stress markers such as malondialdehyde and 8-hydroxy-2’-deoxyguanosine (8-OHdG) are significantly elevated in CKD patients (Kisic et al., 2016; Lopes et al., 2018). ROS production exceeds the body’s antioxidant capacity and prevents it from effectively scavenging ROS, leading to the progression of oxidative stress that damages renal tissues (Olszewska-Czyz et al., 2022; Cao et al., 2024; Wei et al., 2024). Periodontal pathogen-induced ROS exacerbates oxidative damage in renal cells by inhibiting the mitochondrial electron transport chain (Zheng et al., 2021; Subhash et al., 2024). Under oxidative stress, mitochondria produce excessive ROS that react with polyunsaturated fatty acids in the renal cytoplasmic membrane in a lipid peroxidation reaction to form lipid hydroperoxides, which disrupts renal cell membrane integrity and fluidity (Colombo et al., 2020; Irazabal and Torres, 2020; Zhigacheva et al., 2023).ROS can disrupt endoplasmic reticulum calcium pump function in renal cells, leading to calcium overload and unfolded protein reactions (Aparicio-Trejo et al., 2025). At the same time, ROS also reacts with intracellular proteins and DNA, leading to oxidative protein modification and DNA damage, interfering with intracellular signaling pathways and gene expression, and thus affecting the metabolism and repair capacity of renal cells, and promoting the progression of renal diseases (Irazabal and Torres, 2020). A mutually reinforcing relationship exists between the systemic inflammatory response and oxidative stress. The inflammatory response further exacerbates oxidative stress in the body, creating a vicious cycle that collectively causes more severe kidney damage (Zheng et al., 2021; He et al., 2024). Inflammatory mediators and oxidative stress products can cause vascular endothelial dysfunction, leading to vasoconstriction and reduced renal blood flow, affecting renal oxygen and nutrient supply and negatively affecting renal function (Cohen-Hagai et al., 2023). In addition, oxidative stress can further regulate renal hemodynamics by affecting neuroendocrine systems, such as the renin-angiotensin-aldosterone system (RAAS), and aggravate renal injury (Liu et al., 2022).

### 3.2 PD affects CKD through microbe-host interactions-oral flora dysbiosis

PD is a chronic inflammatory disease caused by dysbiosis of the oral flora. Dysbiosis of oral flora leads to the proliferation of pathogenic bacteria in the oral cavity, and these pathogenic bacteria trigger a local inflammatory response that releases large amounts of pro-inflammatory cytokines, chemokines, and tissue remodeling matrix metalloproteinase-like enzymes (MMPs), leading to destruction of periodontal tissues and gradual loss of tooth-supporting structures (Ganther et al., 2021). Oral dysbiosis is not only limited to the localized oral cavity but also triggers a systemic inflammatory response. These inflammatory factors can be transmitted via blood circulation to various organs throughout the body, including the kidneys. The systemic inflammatory response exacerbates kidney damage and promotes the progression of CKD (Makkar and Sriram, 2025). In a controlled study, 100 patients with CKD and healthy patients were recruited, and their saliva was sequenced to analyze the composition of oral microorganisms, revealing a high diversity of oral microorganisms and a significant enrichment of periodontal pathogens in CKD patients (Duan et al., 2020). For example, *Clostridium difficile* infection can cause periodontitis. Induction of periodontitis promotes renal cortical proximal tubule epithelial cell fibrosis and epithelial-mesenchymal transition (EMT) through activation of the TGF-β/SMAD2/3 and β-collagen signaling pathways, which promotes renal mesenchymal fibrosis in rats (Chen et al., 2024). Li et al. fed P. gingivalis to mice to induce macrophage polarization and upregulation of inflammatory factors, which triggered activation of the NF-κB/NLRP3 pathway and iron death in glomerular mesangial cell (GMC) and induced CKD in mice (Li et al., 2024). Genetic sequencing of saliva from patients with CKD compared to healthy participants in a case-control study identified an increase in the oral pathogenic bacteria *lautropia* and *pseudomonas* in patients with CKD and a role for these pathogenic bacteria in kidney injury (Liu et al., 2022). Moreover, changes in the flora of oral microorganisms can also affect the function of transplanted kidneys in CKD, and significant differences in salivary organisms can be an early predictor of the onset of delayed graft function after kidney transplantation (Xiang et al., 2024). Periodontitis caused by oral flora dysbiosis can not only affect CKD, but some specific oral pathogenic bacteria can also cause acute kidney injury. Wei Wei et al. found that periodontitis caused by *porphyromonas gingivals* infection could activate the tumor suppressor M and gingival proteinase, reduce the expression of tight junction protein in the kidney, and lead to the aggravation of acute kidney injury (AKI). Oral dysbiosis may also affect the balance of the intestinal flora through the oral-intestinal axis. Periodontal pathogens may increase the renal burden of the enterogenous toxin indolephenol sulfate by disrupting the intestinal flora (Chang et al., 2017; Choi et al., 2022). Studies have shown that salivary microorganisms from patients with PD can be transferred to the gut, leading to intestinal dysbiosis. Intestinal dysbiosis can further exacerbate the systemic inflammatory response and affect renal function via the gut-renal axis (Bao et al., 2022). For example, one study found that the composition of intestinal flora in stool samples from patients with severe periodontitis differed significantly from that of healthy populations, suggesting that oral dysbiosis may indirectly affect renal health by influencing intestinal flora (Bao et al., 2022).To alleviate the impact of PD on CKD, the balance of oral and intestinal flora can be restored by modulating microbial-host interactions. For example, antibiotics and probiotics can regulate the balance of flora in the body, making it a safe and effective approach by alleviating PD and slowing the progression of CKD (Gao et al., 2022; Kuraji et al., 2024).

### 3.3 Metabolic and endocrine disorders caused by the interaction between PD and CKD- vitamin D deficiency

Abnormal vitamin D (VD) metabolism and periodontitis in patients with CKD form a vicious circle, with low VD levels weakening periodontal tissue repair and exacerbating renal osteodystrophy. Patients with CKD often have disorders of VD metabolism, and the kidneys are an essential site of VD activation; renal function is impaired in CKD, resulting in a decreased ability to convert 25-hydroxyvitamin D (25(OH)D) to 1,25-dihydroxyvitamin D (1, 25(OH)_2_D) (Fusaro et al., 2021; Yeung et al., 2023; Kirkwood et al., 2024). Active VD is essential for maintaining the health of periodontal tissues, which can regulate calcium and phosphorus metabolism, promote periodontal tissue mineralization and repair, and enhance the anti-inflammatory capacity of periodontal tissues (Jørgensen et al., 2024). Low serum VD levels in patients with CKD were associated with the development of chronic periodontitis in a clinical case-control study (Bastos Jdo et al., 2013). VD deficiency in CKD patients weakens the defense mechanisms of periodontal tissues and increases the risk and severity of PD. Disorders of mineral metabolism, such as hyperphosphatemia, hypocalcemia, and elevated levels of parathyroid hormone (PTH), are often seen in patients with CKD (Fusaro et al., 2021; Wang et al., 2024). These metabolic disorders affect the mineralization process of periodontal tissues, leading to periodontal osteoporosis and increasing the susceptibility of periodontal tissues to inflammation, thus aggravating the progression of PD (Fusaro et al., 2021; Wang et al., 2024). In addition, VD has immune-regulating and anti-inflammatory effects, and VD deficiency weakens the immune defenses of periodontal tissues and reduces their resistance to periodontal pathogens (Milan et al., 2022; Kirkwood et al., 2024). VD deficiency may lead to renal calcium and phosphorus metabolism disturbances, further exacerbating abnormal mineral metabolism in CKD patients and promoting renal fibrosis and deterioration of renal function (Vervloet et al., 2023; Yeung et al., 2023). In the interaction between PD and CKD, VD deficiency exacerbates inflammation and oxidative stress, worsening PD and CKD (Milan et al., 2022; Vervloet et al., 2023). VD deficiency is also a key factor in the development of PD and CKD; it exacerbates the vicious cycle between the two. VD supplementation may also effectively treat PD and CKD. In a clinical study by Abel Esteves Suarez et al., eGFR was improved in patients with CKD by supplementation with cholecalciferol (Soares et al., 2017). This was attributed to VD’s anti-inflammatory and antifibrotic effects, which can slow the progression of CKD by blocking the RAAS system. Combining VD and angiotensin-converting enzyme inhibitors/angiotensin receptor blockers slowed CKD progression. Monitoring serum VD levels and periodontal status in patients with CKD may have synergistic preventive value in clinical practice.

## 4 The interaction effect of modifying factors on the association between PD and CKD

### 4.1 The mediating role of cardiovascular disease in PD and CKD

Cardiovascular disease (CVD) may be one of the key mediating factors in the association between PD and CKD. Periodontitis may exacerbate systemic inflammatory responses by releasing inflammatory factors and bacterial toxins into the bloodstream (Kopić et al., 2019; Priyamvara et al., 2022). This systemic inflammatory state damages vascular endothelial function and promotes the formation of atherosclerosis, driving the onset of CVD (Santos-Paul et al., 2019; Ademowo et al., 2020).CVD is the leading cause of death in CKD patients, and periodontitis indirectly affects the cardiovascular prognosis of CKD patients by promoting vascular lesions (Ademowo et al., 2020; Kusumi et al., 2022).CVD can also cause insufficient renal perfusion through hemodynamic changes, thereby exacerbating the progression of CKD (Imamura-Uehara et al., 2024). Mineral and bone metabolism disorders commonly seen in CKD patients can lead to vascular calcification, which is an important mechanism for cardiovascular complications in patients with CKD (Brown, 2019; Kusumi et al., 2022).CVD may play a bidirectional mediating role between PD and CKD and influence the progression and prognosis of CKD.

### 4.2 Synergistic effects of obesity on PD and CKD

Obesity is a risk factor for CKD and is also closely related to PD. Obesity enhances the inflammatory association between PD and CKD (Arinsoy et al., 2016; Rodríguez-Rodríguez et al., 2021; Kanbay et al., 2023). Obesity and PD both cause chronic inflammation in the body, and when they coexist, they may amplify the inflammatory effect and aggravate kidney damage (Kurt-Bayrakdar et al., 2021). Rat experiments have shown that when obesity and periodontitis coexist, inflammatory stress and cellular apoptosis changes in the kidneys are more severe than in either disease alone (Kurt-Bayrakdar et al., 2021). Furthermore, lipopolysaccharides (LPS) from periodontal pathogens can exacerbate damage to renal tubular epithelial cells in a high-fat environment (Chen et al., 2017; Chen et al., 2021). Obesity induced gut microbiota dysbiosis may increase the production of uremic toxins, while the resulting oral microbiota dysbiosis exacerbates systemic inflammation, jointly promoting CKD and PD (Randall et al., 2025). Obesity affects periodontal and renal health through systemic metabolic abnormalities and enhanced inflammatory responses, strengthening the link between PD and CKD.

### 4.3 The enhancing effect of diabetes on the association between PD and CKD

Diabetes is a common risk factor for PD and CKD (Suzuki et al., 2021; Tao et al., 2024). And PD is considered to be one of the complications of diabetes (Kudiyirickal and Pappachan, 2024). The severity of PD in diabetic patients is directly related to poor blood sugar control (Hasan et al., 2021; Kudiyirickal and Pappachan, 2024). Diabetes exacerbates periodontal inflammation by enhancing the virulence and invasiveness of oral pathogens in a hyperglycemic environment, while weakening the host’s immune response and defenses (Li et al., 2021; Kudiyirickal and Pappachan, 2024). This chronic inflammatory state may accelerate the progression of CKD through systemic inflammatory mediators (Kaymaz et al., 2024). Studies have shown that patients with diabetes and periodontitis have a higher risk of CKD, and periodontitis treatment can reduce the risk of dialysis in diabetic patients by 32%–44% (Kusama et al., 2025). Diabetes promotes periodontal tissue destruction and glomerular damage through mechanisms such as oxidative stress and the accumulation of advanced glycation end products (AGEs) (Salimi and Sarrafzadeh Zargar, 2018; Li et al., 2021).In hemodialysis patients, diabetes further exacerbates the severity of periodontitis and the risk of tooth loss, while periodontitis may accelerate the progression of diabetic kidney disease (DKD) through inflammatory responses (Mahajan et al., 2021; Mikami et al., 2022; Liang et al., 2024; Yan et al., 2024). The pathogens responsible for periodontitis can spread to the kidneys via the bloodstream, triggering an inflammatory response within the kidneys. The chronic hyperglycemic state in diabetic patients exacerbates this process, leading to renal microvascular lesions (Karalliedde et al., 2022; Yan et al., 2024). The systemic inflammation caused by periodontitis can damage vascular endothelium, while diabetes itself already involves endothelial dysfunction, and the two synergistically accelerate renal fibrosis (Poznyak et al., 2022; Chapple et al., 2025). Diabetes reinforces the association between PD and CKD through multiple mechanisms, and early control of blood glucose and periodontal inflammation may be significant in preventing or delaying the progression of CKD.

## 5 Clinical interventions for targeted therapy of PD to treat and delay CKD development

**Figure 2:**
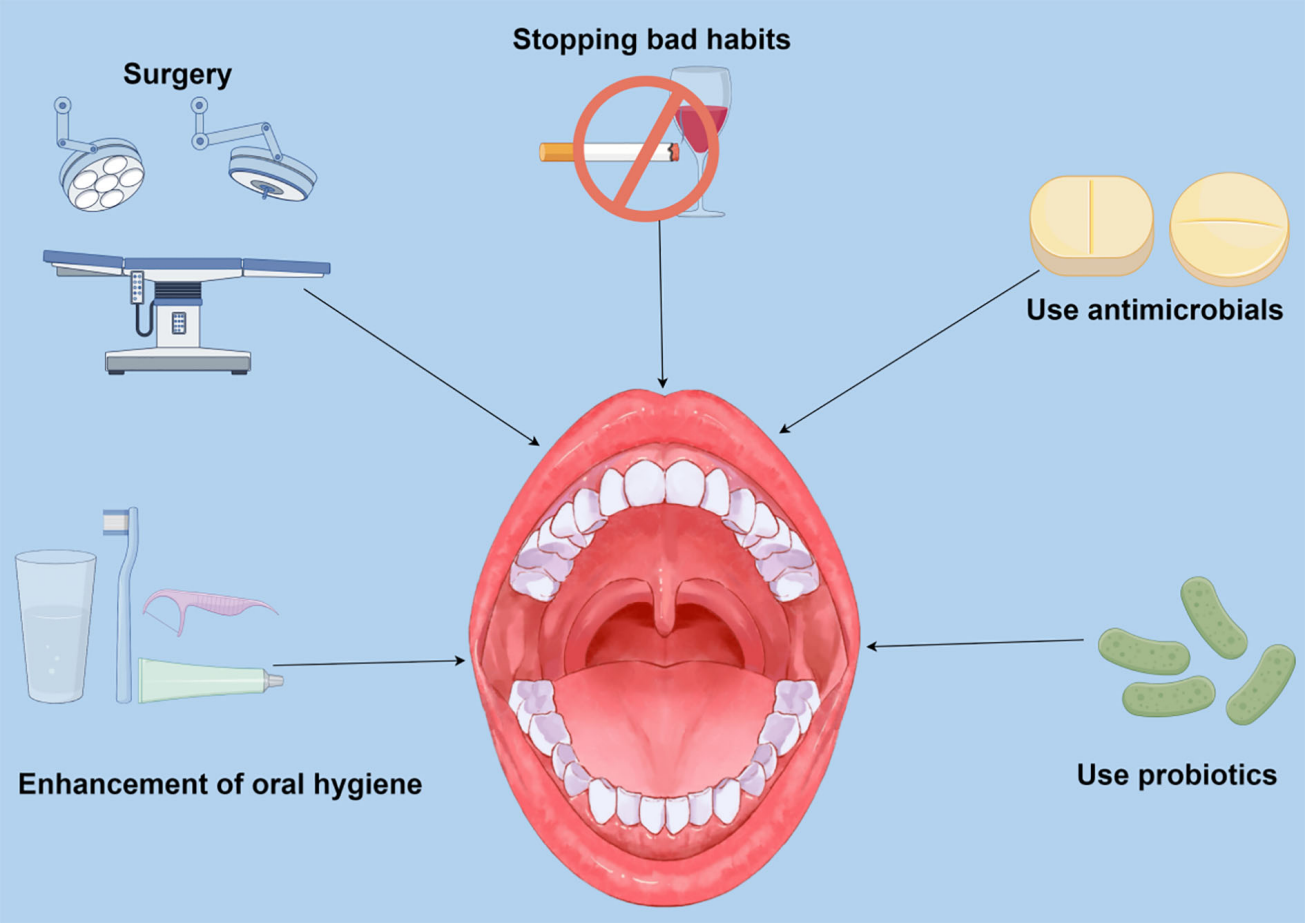
Potential preventive and therapeutic approaches are directed at periodontal disease (PD) caused by oral flora dysbiosis. Current research suggests that targeting PD and modulating oral flora composition to modulate renal status and slow chronic renal progression is a promising therapeutic approach. Smoking and alcohol abuse are recognized as a significant threat to PD, and inflammatory factors produced by oral pathogenic bacteria and PD can affect kidney health in several ways. Recent studies have shown that inhibiting the proliferation of oral pathogenic bacteria and reducing the risk of developing periodontitis by enhancing oral hygiene can impact patients with chronic kidney disease (CKD). Surgical treatment of severe PD mitigates kidney damage by removing necrotic tissue and controlling periodontal inflammation. In addition, antibiotics and probiotics can improve PD by directly altering the composition of the oral microflora and the abundance of specific bacteria.

The occurrence of PD is chronic inflammation mediated by the oral microbiota. The oral pathogenic microorganisms and inflammatory mediators in the blood damage the kidney and aggravate the progression of CKD. In patients with CKD, through some interventions, removal of dental plaque and tartar, periodontal surgery, antimicrobial drugs and antibacterial mouthwash, and use of oral probiotics. These methods of treating PD to delay the development of CKD should be considerable in preventing and treating CKD (**Figure 2**).

### 5.1 Stopping bad habits

Poor lifestyle habits directly and profoundly affect oral health and kidney health. Maintaining a healthy lifestyle and stopping bad habits can help regulate the homeostasis of the oral microbiota and reduce the risk of PD, thereby preventing and treating CKD. Smoking is a well-defined risk factor for periodontal disease. It exacerbates periodontal inflammation through mechanisms such as interfering with the host immune response, altering the subgingival microbial community, and impairing the ability of tissues to heal (Apatzidou, 2022; Zhang et al., 2024). In a systematic evaluation study by Jenny Zhang et al., smokers who underwent nonsurgical periodontal treatment showed significantly less reduction in pocket depth and improved clinical attachment level than nonsmokers (Chang et al., 2021). In a longitudinal study in Japan that included 1,332 adult men, smoking was found to be an independent risk factor for PD and tooth loss (Okamoto et al., 2006). Alcohol consumption, especially alcohol abuse, is associated with an increased risk of PD, and the effect is more pronounced in low-income populations (Oliveira et al., 2024). Smoking has also been linked to the development and progression of chronic kidney disease. In a study based on a database of British samples, it was found that the shorter the time between waking up and smoking the first cigarette, the higher the risk of chronic kidney disease (Tang et al., 2024). And in another Korean cohort study, the risk of worsening kidney function was significantly higher with increasing cigarette consumption over the years (Lee et al., 2021). Smoking duration was linearly associated with decreased renal function, and the risk of adverse renal outcomes decreased with more extended periods of abstinence (Lee et al., 2021). Chronic alcohol abuse exacerbates the localized periodontal inflammatory response and increases systemic oxidative stress through hepatic metabolites, further impairing renal function (Pinto et al., 2024). In a population-based cohort study that included 1,285 participants, by recording alcohol intake and following up on the periodontal condition of the patients, the results of the study found that higher alcohol intake increased the risk of periodontitis (Baumeister et al., 2025). A controlled experiment using alcohol and nicotine-induced rats by Kareem-Paula Pinto et al. showed that combined exposure to alcohol and nicotine significantly exacerbated bone destruction in a model of periapical periodontitis and elevated serum inflammatory factors (TNF-alpha, IL-6) and accelerated renal fibrosis (Pinto et al., 2024). Chronic alcohol intake alters the oral microbial composition and increases proinflammatory cytokine production, leading to the destruction of periodontal bone microstructure (Pinto et al., 2020; Pinto et al., 2024). Alcoholism also causes direct damage to the kidneys. In a 12-year study from the China Health and Nutrition Examination Survey, researchers found that excessive drinking >18 standard drinks/week significantly increased the risk of CKD (Li et al., 2022b). And in a cross-sectional study of 3,374 participants, it was found that occasional alcohol consumption may increase the risk of stage 3–4 CKD compared to non-drinkers (Moeinzadeh et al., 2023). Reducing alcohol intake improves periodontal microcirculation and decreases serum C-reactive protein (CRP) levels, thereby delaying the decline in glomerular filtration rate in patients with CKD (Baumeister et al., 2021; Baumeister et al., 2025). Smoking and alcohol consumption create a vicious cycle with CKD by exacerbating periodontal inflammation and systemic oxidative stress. Improving lifestyle habits such as smoking cessation, alcohol restriction, and active treatment of PD may be critical adjunctive strategies for managing patients with CKD.

### 5.2 Enhancement of oral hygiene

PD is a chronic inflammatory disease caused primarily by plaque biofilm accumulation, manifesting as gingivitis and periodontal tissue destruction. Conventional treatment has focused on mechanical plaque control and enhanced oral hygiene, with key interventions including brushing, flossing, and the use of antimicrobial mouthwashes and antimicrobial toothpaste (Jönsson and Abrahamsson, 2020; Steinberg and Friedman, 2020). Regular brushing and flossing reduce plaque formation and disrupt the formation of disease-causing biofilms, thereby reducing gingival inflammation and lowering the risk of periodontal tissue destruction (Parsegian et al., 2022). The new AI-assisted multimodal sensor toothbrush guides patients in real-time to optimize brushing behavior and improve compliance, and helps clinicians develop personalized intervention plans through data transfer (Li et al., 2024). Using a mouthwash or toothpaste containing green tea polyphenols may improve gum health through anti-inflammatory and antioxidant effects (Shimizu et al., 2023). Annual periodontal cleanings and exams allow for early detection and intervention of periodontal lesions and reduce the formation of deep periodontal pockets (Hirai et al., 2020). Improving oral hygiene to treat periodontitis effectively prevents PD and is a key strategy to interrupt the vicious cycle of periodontitis and CKD.

### 5.3 Surgical treatment of PD

Periodontal surgery to treat PD is an essential clinical intervention for moderate to severe periodontitis, with the core goal of restoring the function and stability of tooth-supporting structures by removing infected tissues and promoting periodontal tissue regeneration. Periodontal surgery is mainly indicated for patients with moderate to severe periodontitis, especially in periodontal pockets ≥5mm deep, intraosseous defects, or root bifurcation lesions (Dommisch et al., 2020; Zhao et al., 2023). Surgical periodontal treatment reduces the systemic inflammatory burden by removing pathogenic bacterial biofilms from periodontal pockets and blocking the entry of bacteria and toxins into circulation through the ulcerated gingival mucosa (Wesson et al., 2022; Makkar and Sriram, 2025). Surgery significantly reduces the levels of proinflammatory factors in the gingival sulcus fluid and serum markers of inflammation; molecules strongly associated with CKD progression (Shimizu et al., 2023). By eliminating the foci of chronic periodontal infections, surgery may improve immune dysregulation in CKD patients, such as reducing oxidative stress damage due to neutrophil overactivation (Sharma et al., 2021).

### 5.4 Antibiotic use

Oral pathogenic bacteria primarily cause PD, and periodontitis is a chronic inflammatory disease triggered by pathogenic bacteria in the plaque biofilm. An imbalance in the healthy oral microbial community is also a central feature of periodontitis. The proliferation of pathogenic bacteria disrupts the host-microbe balance, forming a pathogenic biofilm dominated by anaerobic bacteria (Larsson et al., 2022; Li et al., 2022a; Tonoyan et al., 2025). Pathogenic bacteria’s persistent activation of immune cells leads to excessive release of proinflammatory factors and reactive oxygen species, triggering chronic inflammation and destruction of periodontal tissues (Yin et al., 2022; Luo et al., 2025; Zhang et al., 2025). Oral pathogens and their products can spread through bloodstream or the oral-intestinal axis diffusion, which is associated with the development of CKD. A practical management approach to PD requires removing pathogens and restoring the oral microbial community balance. Antibiotics directly kill pathogenic bacteria in the oral cavity, such as *Aggregatibacter* and *Porphyromonas gingivalis*, and penetrate the biofilm formed by the pathogenic bacteria to enhance the bactericidal effect. Amoxicillin, combined with metronidazole, a standard regimen for aggressive periodontitis, significantly changes the number of oral pathogenic bacteria over time (Cosgarea et al., 2022; Hagenfeld et al., 2023). However, antibiotic treatment modalities are usually used as adjunctive therapy after periodontal surgery, and for short-term use to control oral pathogens; systemic long-term use is not recommended. An animal study by Lei Zhu et al. found that intestinal dysbiosis induced by chronic exposure of mice to systemic antibiotics disrupts oral flora and exacerbates periodontitis and oral bone loss (Yuan et al., 2023). Prolonged systemic use of antibiotics may lead to dysbiosis of gut ecology, systemic side effects, and increased risk of drug resistance (Ng et al., 2024). Some studies have pointed out that antibiotics may disrupt the microecological balance of the oral cavity, which in turn affects the long-term therapeutic effects (Yuan et al., 2023; Ng et al., 2024). Microorganisms in periodontal pockets are susceptible to resistance to commonly used antibiotics and need to be carefully selected for administration (Tonon et al., 2023; Ng et al., 2024).

### 5.5 Oral probiotic

Probiotics as an adjunctive treatment for PD have received widespread attention recently. Their mechanism of action mainly includes regulating the ecological balance of the oral microbial community, inhibiting pathogenic bacterial colonization, modulating the host immune response, and attenuating the inflammatory response. The development of PD is closely related to the dysregulation of the oral microbial community. Probiotics such as *Lactobacillus* and *Bifidobacterium* can restore the oral microecological balance by competitively inhibiting the colonization of periodontal pathogens (Grilc et al., 2023; Hu et al., 2025). For example, bacteriocins produced by *Lactococcus lactis* significantly reduce the number of periodontal pathogens and decrease alveolar bone resorption (Hu et al., 2025). The use of probiotics in treating PD has been shown to reduce the risk of PD. In addition, probiotics may reduce periodontal inflammation by modulating the host immune response. Clinical studies have found that probiotics can reduce the levels of pro-inflammatory cytokines (TNF-α, IL-1β) while enhancing the anti-inflammatory response (Balta et al., 2021; Luo et al., 2024). For example, *Lactobacillus rhamnosus* can enhance the host’s defensive immune response by modulating Cluster of Differentiation 14 (CD14) expression, thereby attenuating tissue damage (Luo et al., 2024). The use of probiotics in periodontal inflammation has also been shown to reduce inflammation in the dentition. Moreover, some probiotics have shown the potential to promote periodontal tissue regeneration. Animal studies have shown that probiotics can improve alveolar bone regeneration by increasing osteogenic metabolites through the gut-blood-bone axis (Moraes et al., 2022). In addition, combining probiotics and nanofibers can enhance antioxidant activity and protect probiotic activity, providing a new idea for functional treatment (Ma et al., 2024). Conventional antibiotic therapy carries the risk of resistance and disruption of normal flora. Probiotics and their metabolites can be used as an alternative to target and regulate periodontal biofilms with a high degree of safety (Ren et al., 2023; Wu et al., 2024). For example, in rat experiments, heat-inactivated *Lactobacillus fermentum* and its supernatant modulated the inflammatory response and attenuated periodontal bone loss, reducing periodontitis symptoms (Ye et al., 2023). Although probiotics have shown potential in periodontal therapy, there is heterogeneity in the results of existing clinical studies, which may be related to strain selection, mode of administration, and individual differences (Ren et al., 2023; Ma et al., 2024). Future studies are needed to optimize probiotic formulations in combination with prebiotics or nanocarriers and to explore their long-term efficacy and impact on systemic health.

## 6 Combined management strategies for PD and CKD

The phenomenon that periodontal disease and CKD can contribute to each other’s disease progression emphasizes the importance of comprehensive treatment, and that treating PD while focusing on and managing kidney function can lead to better patient outcomes. A thorough patient assessment includes periodontal health status and renal function indicators. A periodic review of periodontal and renal-related indices is required to detect changes in the condition and adjust the treatment plan promptly. For patients with CKD, periodontal examination every 3–6 months is recommended, along with regular monitoring of renal function, depending on the severity of kidney disease (Hasegawa and Nangaku, 2022; Davison et al., 2024). The examination should include periodontal pocket depth, attachment loss, and tooth loss. Renal function assessment involves testing serum creatinine, estimated glomerular filtration rate (eGFR), and urinary albumin (Le, 2021; Villoria et al., 2024). Periodontal treatments include scaling and root planning to remove plaque and tartar and reduce periodontal inflammation. These treatments can reduce periodontal inflammation and decrease systemic inflammation levels, which may benefit kidney function in patients with CKD (Alkakhan et al., 2023; Luan et al., 2023). For severe PD, periodontal surgery may be necessary. Surgical treatment can further control periodontal inflammation and improve the health of periodontal tissues (Luan et al., 2023). Adjunctive therapeutic measures such as antimicrobial drugs and mouthwashes can enhance the therapeutic effect. At the same time, probiotics can be used to regulate oral microecology and reduce the growth of harmful bacteria (Gao et al., 2022; Alkakhan et al., 2023). A good lifestyle can help control CKD’s progression and reduce risk factors for PD (Goraya et al., 2024). Lifestyle interventions mainly include smoking cessation and limiting alcohol consumption. In patients with advanced CKD, such as those with renal failure, renal replacement therapy, including hemodialysis, peritoneal dialysis, or renal transplantation, may be required. Oral hygiene should be observed during these treatments to minimize the risk of infection (Hasegawa and Nangaku, 2022). Dentists and nephrologists should work closely together to develop a treatment plan for the patient. When performing periodontal therapy, dentists need to consider the patient’s renal function status and avoid medications that are potentially damaging to the kidneys; nephrologists, on the other hand, should pay attention to the patient’s oral health status and make timely referrals to the dentist for periodontal therapy (Villoria et al., 2024; Chapple et al., 2025).

## 7 Conclusion and future directions

Further in-depth studies are needed to investigate the underlying mechanisms by which PD and CKD interact, including inflammatory responses, oxidative stress, immune system activation, and microbial community changes. Although current research has revealed an association between PD and CKD, the causality of this association has not been fully clarified. More large-scale, long-term prospective cohort studies and randomized controlled trials are needed to determine whether PD is an independent risk factor for CKD and whether CKD affects the onset and progression of PD. New research directions could be used for early detection and intervention of diseases by looking for biomarkers that predict the progression of PD and CKD. For example, studying potential biomarkers in the gingival sulcus fluid and serum could inform personalized medicine. There is a need to assess the long-term effects of different periodontal treatment interventions on inflammation levels, oxidative stress markers, renal function, and cardiovascular disease risk in CKD patients to provide a more substantial basis for clinical management. New therapeutic approaches, such as circulating RNA-based therapeutic strategies, are also being explored to improve the prognosis of patients with PD and CKD. The influence of different individual factors on the interrelationship between PD and CKD and the presence of specific at-risk populations should be explored to develop more precise prevention and treatment strategies. Considering the association of PD and CKD with other chronic diseases such as diabetes, cardiovascular disease, and obesity, future studies could further explore the interaction networks between these diseases and the role and place of PD in managing chronic diseases.
